# New progress of glutamine metabolism in the occurrence, development, and treatment of ovarian cancer from mechanism to clinic

**DOI:** 10.3389/fonc.2022.1018642

**Published:** 2022-11-29

**Authors:** Xiaojing Yang, Zhen Li, Hanru Ren, Xue Peng, Jie Fu

**Affiliations:** ^1^ Department of Radiation Oncology, Shanghai Sixth People's Hospital Affiliated to Shanghai Jiao Tong University School of Medicine, Shanghai, China; ^2^ Department of Orthopedics, Shanghai Pudong Hospital, Fudan University, Pudong Medical Center, Shanghai, China; ^3^ Department of Breast Surgery, Shanghai Sixth People's Hospital Affiliated to Shanghai Jiao Tong University School of Medicine, Shanghai, China

**Keywords:** ovarian cancer, glutamine (Gln), mechanism, resistance, metabolism

## Abstract

Glutamine is a non-essential amino acid that can be synthesized by cells. It plays a vital role in the growth and proliferation of mammalian cells cultured *in vitro*. In the process of tumor cell proliferation, glutamine not only contributes to protein synthesis but also serves as the primary nitrogen donor for purine and pyrimidine synthesis. Studies have shown that glutamine-addicted tumor cells depend on glutamine for survival and reprogram glutamine utilization through the Krebs cycle. Potential therapeutic approaches for ovarian cancer including blocking the entry of glutamine into the tricarboxylic acid cycle in highly aggressive ovarian cancer cells or inhibiting glutamine synthesis in less aggressive ovarian cancer cells. Glutamine metabolism is associated with poor prognosis of ovarian cancer. Combining platinum-based chemotherapy with inhibition of glutamine metabolic pathways may be a new strategy for treating ovarian cancer, especially drug-resistant ovarian cancer. This article reviews the role of glutamine metabolism in the biological behaviors of ovarian cancer cells, such as proliferation, invasion, and drug resistance. Its potential use as a new target or biomarker for ovarian cancer diagnosis, treatment, and the prognosis is investigated.

## 1 Introduction

Ovarian cancer has the highest mortality rate in women, causing an estimated 140,000 people worldwide to die each year (1). Currently, ovarian cancer treatment mainly includes surgery, chemotherapy, molecular targeted therapy and hormone therapy, etc. (2). Since the deep location of the ovaries in the pelvis prevents early symptoms from being noticeable, making the clinical diagnosis difficult, the vast majority of ovarian cancer patients are diagnosed at an advanced stage. Moreover, ovarian cancer is prone to metastasis and recurrence, which has become a tricky hot spot in gynecological cancer research (3). Therefore, it is crucial to find the causes of ovarian cancer progression and select novel biomarkers as target in treating ovarian cancer.

Glucose and glutamine (Gln) are the primary energy sources for the rapid proliferation of ovarian cancer cells, especially Gln (4). The activity of glutaminase (GLS) is significantly increased in cancer patients. GLS is a rate-limiting enzyme of Gln catabolism and is closely tied to tumor growth, angiogenesis, and immunity (5). Gln is an abundant and non-essential amino acid in the human body. Gln synthesis cannot meet cell proliferation needs in fast-proliferating tumor cells such as ovarian cancer cells. Gln is then converted into a conditionally essential amino acid. Glutamine metabolic enzymes and the overexpression of glutamine transporter lead to glutamine uptake and utilization under the regulation of oncogenes. Through this procedure, tumor cells create a vital energy and metabolic exchange (6).. Studies have shown that Gln can enter ovarian cancer cells through selective amino acid transporters, and plays a vital role in the occurrence, metastasis, invasion, and treatment of ovarian cancer (7). Blocking Gln tricarboxylic acid cycle in highly aggressive ovarian cancer cells or inhibiting Gln synthesis in less aggressive ovarian cancer cells may be a potential treatment for ovarian cancer. Inhibition of Gln pathways and platinum-based combination may be a new strategy for the treatment of ovarian cancer, especially drug-resistant ovarian cancer (8). Since the current summaries of Gln metabolism in ovarian cancer are limited, this article reviews the research progress of Gln metabolism as well as the ovarian cancer occurrence and development, provides new treatment strategies for ovarian cancer patients and explores the ovarian cancer mechanisms from the perspective of metabolism.

## 2 Related signals and genes regulating Gln metabolism in ovarian cancer

Tumor metabolism is dominated by glycolysis. The accumulation of lactic acid produced by glycolysis leads to the acidic state of the tumor immune microenvironment. A low pH microenvironment can affect T cell function, promote the immune escape of tumor cells, and promote tumor occurrence, development, and metastasis (9). Genes involved in the regulation of Gln metabolism include mTORC1, myc, and phosphatidylinositol 3-kinase (PI3K) (4, 10, 11).

### 2.1 mTORC1

As an amino acid sensor, mTOR is indirectly or directly involved in the regulation of cellular metabolism (12). One of its forms, mTORC1, transfers extracellular Gln into cells in the absence of amino acids (13). In ovarian cancer cells, mTORC1 is closely associated with Gln metabolism, and the mTORC1 signaling pathway is inhibited if the expression of the glutamine transporter is silenced (14). Pusapati et al. found that combined inhibition of glycolysis and mTORC1 signaling disrupts metabolic reprogramming in ovarian cancer cells and inhibits their growth *in vitro* and *in vivo* (15).

### 2.2 Hypoxia-inducible factor 1

HIF-1 is a crucial transcription factor produced by tumor cells under hypoxic conditions. It is closely related to angiogenesis and glycolysis (16). HIF-1 participates in the TCA cycle and the synthesis of citric acid to promote Gln metabolism in a hypoxic state (17). HIF-1α also increased the expression of GLS in cells (18). HIF-1-based research can explore and discover corresponding targets affecting ovarian cancer metabolic reprogramming and energy metabolism. Studies have found that the reduced expression of mitochondrial coenzyme I (NAD)-dependent deacetylase SIRT3, a vital regulator of the Warburg effect, can regulate reactive oxygen species production, the increase of HIF-1 expression, and Gln metabolism regulation (19). Nakayama et al. found that the HIF-1α gene up-regulated the expression of VEGF in ovarian cancer and was involved in the angiogenesis of ovarian cancer (20). Tumor cell proliferation was inhibited in ovarian cancer when HIF-1 expression was inhibited (21).

### 2.3 PI3K

PI3K is an essential factor affecting cell proliferation, differentiation, and apoptosis. In ovarian cancer, PI3K/Akt is abnormally activated, and the utilization of Gln is significantly enhanced (22). Studies have found that HIF-1α silencing can attenuate the viability of hypoxic ovarian cancer cells and increase apoptosis and autophagy through the PI3K/AKT/mTOR signaling pathway (23).

### 2.4 myc

The proto-oncogene, myc, can promote mitochondrial Gln glycolysis in tumor cells, and the upregulation of myc is the cause of Gln addiction in tumor cells (24). Myc completes this process by upregulating key enzymes or glucose and glutamine transporters in the glutaminolysis pathway, as well as glycolysis. It also coordinates cellular utilization of glucose and Gln in biosynthetic pathways by directly regulating mitochondrial mass and activity (25). Li et al. found that miR-145 inhibits glutamine metabolism by targeting c-myc and c-myc can promote GLS1 expression through transcriptional activation (4). Their research suggests that myc mainly serves as an antioxidant regulating the downstream of GLS1.

### 2.5 STAT3

As a signal transducer and activator of transcription, STAT3 induces the rewiring of metabolic pathways by participating in cell differentiation, anti-apoptotic responses, metastasis, and large-scale signaling systems (26). ERK1/2 and Janus-activated kinase 1 (JAK1) affect cancer cell proliferation, metabolic regulation, and cancer cell metastasis through the regulation of STAT3 amino acid phosphorylation levels in invasive ovarian cancer cells (27). Therefore, continuous activation of STAT3 can induce myc and cyclin D and inhibit the expression of BclXL, facilitating the growth and survival of tumor cells. In highly aggressive ovarian cancer cells, Gln breaks down into the TCA cycle relying on GLS and activates JAK1. JAK1 further activates STAT3 through tyrosine phosphorylation to regulate glycolysis. In other words, Gln breakdown increases the invasiveness of ovarian cancer cells (28). In addition, Gln also promotes STAT3 serine phosphorylation by activating ERK1/2 and the interaction with mitochondrial complexes I and II enhances oxidative phosphorylation within mitochondria. This process increases the TCA cycling activity of OVCA cells, and further increases the invasiveness (29).

### 2.6 JAK-STAT

The JAK-STAT signaling pathway can regulate the expression of the downstream oncogene myc and promote the cell transformation during the G1 phase and S phase, cell proliferation and tumor transformation (30). It has been reported that myc can induce the expression of mitochondrial and promote the catabolism Gln by inhibiting miR-23a and miR-23b (31). Studies have confirmed that the catabolism of Gln is associated with high invasiveness and poor prognosis of ovarian cancer cells. Therefore, the JAK-STAT signaling pathway can indirectly regulate the expression of glutaminase through the oncogene myc and play an important role in the metabolism of ovarian cancer cells. At the same time, the decomposition of Gln can positively activate the expression of the JAK-STAT signaling pathway. The positive feedback enhances the invasiveness of ovarian cancer cells. Moreover, myc can induce cisplatin resistance in ovarian cancer cells (32).

**Figure 1:**
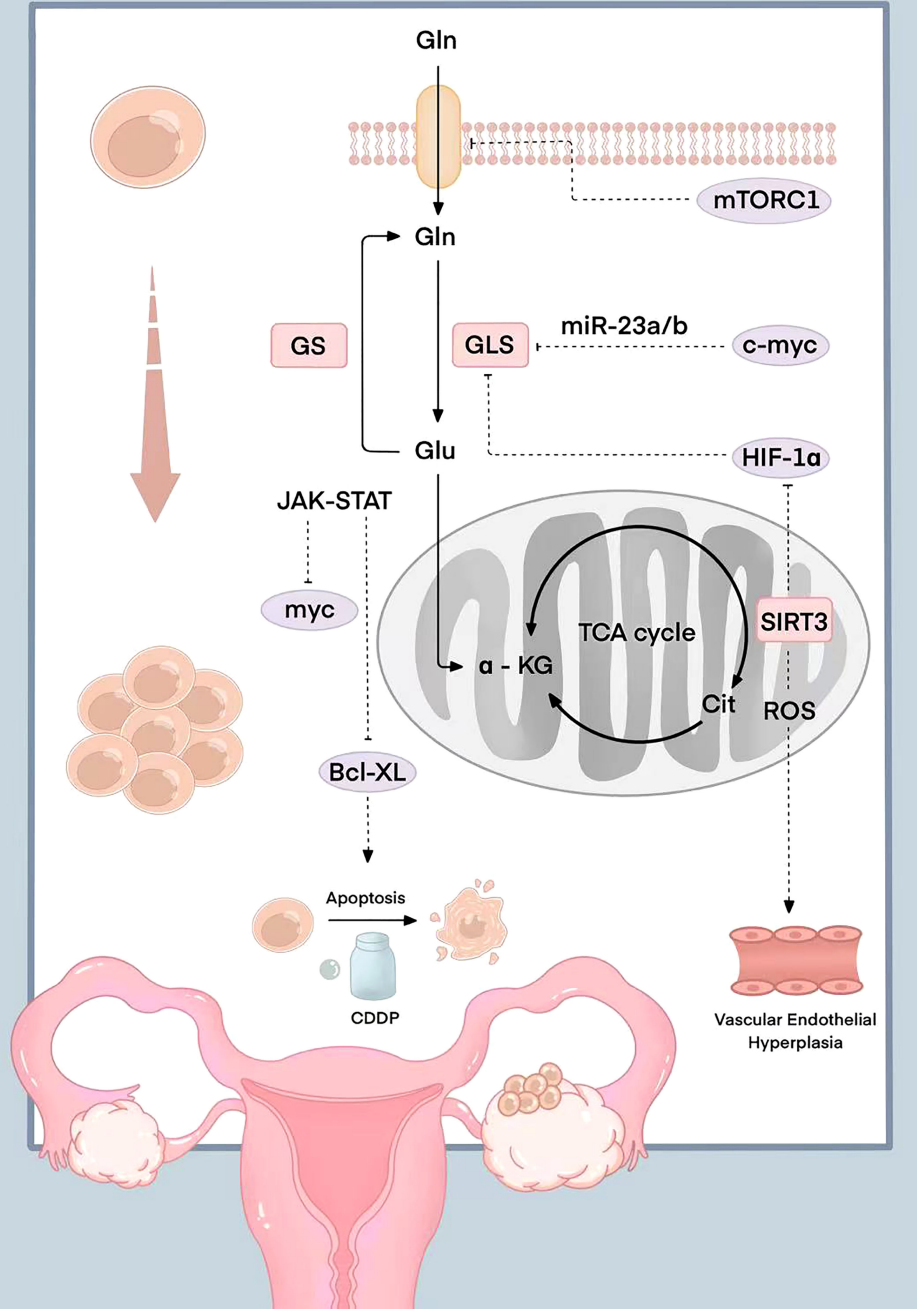
Schematic diagram of the molecular mechanism related to Gln in ovarian cancer. mTORC1 is inhibited when the expression of the glutamine transporter is silenced; myc induce the expression of mitochondrial and promote the catabolism Gln by inhibiting miR-23a/b; HIF-1 participates in the TCA cycle and the synthesis of citric acid to promote Gln metabolism in a hypoxic state; The reduced expression of mitochondrial coenzyme I (NAD)-dependent deacetylase SIRT3 can regulate reactive oxygen species production, the increase of HIF-1 expression, and Gln metabolism regulation; HIF-1α up-regulated the expression of VEGF and was involved in the angiogenesis; The JAK-STAT signaling pathway activated by Gln decomposition can regulate the expression of anti-apoptotic gene Bcl-XL.

The JAK-STAT signaling pathway activated by Gln decomposition can also regulate the expression of the downstream anti-apoptotic gene Bcl-XL. The high expression level of Bcl-XL makes ovarian cancer cells resistant to programmed apoptosis caused by chemotherapy drugs. Stover et al. found that ovarian cancer cells with high Bcl-XL expression were highly resistant to cisplatin and paclitaxel. The drug resistance of ovarian cancer cells was positively correlated with Gln catabolism (33). However, Gln doesn’t directly interact with a kinase or a transcription factor to alter its function. The relevant molecular mechanism is shown in **Figure 1**.

## 3 The role of Gln metabolism in the clinical course of ovarian cancer

### 3.1 Gln promotes the growth of ovarian cancer cells

Gln is one of the most important nutrients for tumor cell biosynthesis. The dependence of ovarian cancer cell lines (HEY, SKOV3, and IGROV-1) on Gln has been assessed by analyzing cytotoxicity, cell cycle progression, apoptosis, cellular stress, and glucose/Gln metabolism. The results show that the proliferation of three cell lines is dose-dependent on Gln (34). Gln can promote cells enter the G1 phase to the S phase. Annexin-V analyzed the effect of Gln on apoptosis and found that the apoptosis rate of ovarian cancer cells in a Gln-deficient medium was significantly increased. The production of ROS and the expression of cell stress-related proteins are induced at the same time. Mitogen-activated protein kinase/extracellular signal-regulated kinases (MAPKs/ERK) and PI3K/AKT/mTOR pathways are critical in controlling the growth and survival of ovarian cancer cells. Gln can increase S6 (Ser235/236) and p42/44 (ERK) phosphorylation, indicating that MAPK/ERK and mTOR/S6 are involved in Gln metabolism. The mTOR inhibitor rapamycin can strongly inhibit the phosphorylation of S6 and the expression of GLS, and Gln promotes the proliferation of ovarian cancer cells through the mTOR/S6 pathway. In addition, Gln increases GLS and glutamate dehydrogenase (GDH) activities by regulating mTOR/S6 and MAPK pathways (34, 35). The above studies suggest that Gln metabolism has a very important role in promoting the growth of ovarian tumors. Therefore, whether it is possible to target key syntheses in the Gln metabolism pathway and seek new treatment options for ovarian cancer deserve in-depth discussion. It is well known that Gln metabolism is critical for cellular reactive oxygen species (ROS) homeostasis (36), and some Gln metabolic pathways can directly regulate ROS levels (37). We should think about whether ovarian cancer carcinogenesis and ROS homeostasis, which is regulated by Gln metabolic pathways, are related.

### 3.2 Gln is associated with ovarian cancer cell invasion

The diverse tumor microenvironments suggest that cells require nutrients for growth, invasion, and energy metabolism. An increasing number of studies have shown that tumor cell metabolism is mainly driven by oncogene alterations and reprogramming of cellular metabolic processes. However, recent cancer invasion and malignancy studies have shown that tumor is metabolism-dependent. Glucose and Gln promote tumor cell growth. The dependence of tumor cell invasion and migration ability on specific tumor cell metabolites remains unclear. Interactions of glucose metabolism and tumor cell survival pathways affect cell invasion, migration, and energy balance. Many targeted drugs for glycolysis regulate cell differentiation, anti-apoptosis, and metastasis by activating epidermal growth factor receptor (EGFR), oncogene Src, JAK, and ERK pathways (38, 39). However, the network of Gln metabolism and control of tumor cell phenotype is still unclear. The metabolic network of ovarian cancer patients showed that patients with poor prognosis have high expression of Gln catabolism-related genes, including GLS1, GDH, aspartate aminotransferase 1 (GOT1) and GOT2, and tricarboxylic acid cycle-related genes [such as Pyruvate dehydrogenase (PDH), citrate synthase (CS), aconitase (ACO2) and succinate dehydrogenase (SDHB)]. This is in contrast to the expression of genes involved in the entry of glucose into the Krebs cycle. Gas chromatography-mass spectrometry (GC-MS) technology was used to analyze the abundance of metabolites related to OVCAR3 and SKOV3 ovarian cancer cell lines by targeted metabolomics. By GC-MS, glycolysis Pathway (pyruvate, lactate) and tricarboxylic acid cycle (citric acid, malic acid, and fumaric acid) metabolites of low-invasive ovarian cancer cells (LI-OVCA) were found to be higher than those in highly aggressive ovarian cancer cells (HI-OVCA). Using isotope tracer technology and bioenergetics analysis, it was found that LI-OVCA was Gln-independent, and HI-OVCA was significantly Gln-dependent, suggesting that Gln metabolism supports mitochondrial tricarboxylic acid cycle activity in highly aggressive ovarian cancer. Therefore, Gln metabolism is associated with poor prognosis in ovarian cancer, rather than glycolysis (29).

By studying the relationship between ovarian cancer cells and glutamine, Yang et al. found that the proliferation of highly invasive ovarian cancer cells is dependent on glutamine. In contrast, the low-invasive ovarian cancer cells use the conversion of glucose metabolism to glutamine to compensate for the deficiency of Gln (35). Absence of this metabolic process results in low Gln-dependence in low-invasive ovarian cancer cells. Some scholars have analyzed ovarian cancer patients’ gene expression and survival data by analyzing the cancer genome atlas (TC-GA). They found that glutamine decomposition can increase the mitochondrial TCA cycle activity in highly aggressive ovarian cancer patients (40). Caneba et al. confirmed that highly aggressive ovarian cancer cells mainly use Gln decomposition products as TCA cycle metabolites instead of pyruvate, thereby promoting tumor cell invasion and metastasis (41). Cheng et al. found that low-invasive ovarian cancer cells can decompose glucose into the TCA cycle through pyruvate carboxylation, thereby exerting mitochondrial activity and promoting tumor invasion and metastasis (42).

### 3.3 The high expression of glutaminase affects the prognosis of ovarian cancer

The studies mentioned above suggest that Gln may play a key role in the growth of highly aggressive ovarian tumors and overall patient survival. Muys et al. found that glutamate dehydrogenase (GLUL) expression was high in low-invasive ovarian cancer cells. The expression of glutaminase, which breaks down Gln, was higher in highly aggressive ovarian cancer cells. By further evaluating the clinical significance of the ratio of GLN catabolism to anabolism, it was found that ovarian cancer patients with high GLS protein expression were associated with lower overall survival compared with patients having high GLUL protein expression (43). The study of glutamine metabolism found that the gene expression ratio related to Gln catabolism and anabolism has become a biomarker for predicting patient prognosis.

### 3.4 Gln and ovarian cancer treatment

#### 2.4.1 Anti-ovarian cancer drugs based on Gln metabolism

Gln metabolism is involved in the occurrence and development of various cancers, giving great application prospects for its targeted drugs. The most studied drugs are glutamine metabolizing enzymes and their transporter inhibitors. **Table 1** shows the relevant Gln metabolized drugs in ovarian cancer.

**Table 1:** Drugs targeting on glutamine metabolism in ovarian cancer.

Class	Drugs	Status
ASCT2 inhibitors	Anti-ASCT2 antibody (44)	Preclinical tool
GLS inhibitors	BPTES (45)	Preclinical tool
	Compound 968 (46)	Preclinical tool
	CB-839 (47)	Preclinical tool
xCT inhibitors	Erastin (48, 49)	Tool compound
	Sorafenib (50, 51)	Approved for anti-cancer
	Sulfasalazine (52)	Approved for arthritis

ASCT2, Alanine-serine-cysteine transporter 2; GLS, glutaminase; BPTES, bis-2-(5-phenylacetamido-1,2,4-thiadiazol-2-yl) ethyl sulfide 3; xCT, cystine/glutamate transporter cystine-glutamate exchange.

##### 3.4.1.1 Alanine-serine-cysteine transporter 2 inhibitors

The expression of ASCT2 (SLC1A5) is significantly increased in various tumors, including ovarian cancer. The loss of its activity or expression can effectively inhibit tumor growth. ASCT2 monoclonal antibody is more specific and stable than traditional small molecule inhibitors and can produce antibody-dependent cytotoxicity. Using Chinese hamster ovary cells expressing SLC1A5 as immunogens, Suzuki et al. generated monoclonal antibodies KM4008, KM4012, and KM4018 that could recognize the ASCT2 cell surface domain. As neutralizing antibodies, these three monoclonal antibodies inhibited glutamine-induced colon cancer cell proliferation in a dose-dependent manner (53). Through molecular processes unrelated to cytotoxic drug sensitivity, SLC1A5 reduces ovarian cancer recurrence in the pathogenesis of the disease (54). Studies have shown that the combined detection of ASCT2 and p-mTOR can be used as a molecular marker for ovarian cancer, providing reference information for the diagnosis, postoperative follow-up, and targeted therapy of early ovarian cancer patients (44).

The small molecule inhibitor L-Glutamyl-p-nitroanilide (GPNA) inhibits glutamine uptake and induces ROS enrichment by binding ASCT2. In addition, V-9302 is a small molecule antagonist developed based on 2-amino-4-2 (aryloxybenzyl) aminobutyric acid, which can bind to ASCT2. It is currently in the preclinical research stage, and it sensitizes CB-839-treated glutamine-dependent (GD) cells (55). However, the use of GPNA and V-9302 has not been reported in ovarian cancer so far.

##### 3.4.1.2 GLS inhibitors

Bis-2-(5-phenylacetamido-1, 2, 4-thiadiazol-2-yl) ethyl sulfide 3 (BPTES) is an allosteric inhibitor of GLS1 (56). It specifically binds to GLS1, inhibits its phosphorylation and activation, and even has an inhibitory effect on GLS1 after phosphorylation activation (57). The study shows that BPTES treatment resulted in a significant reduction in the ability of glutamine-dependent ovarian cancer cells to form colonies in a clonogenic assay. BPTES resensitizes paclitaxel- and cisplatin-resistant ovarian cancer cells to chemotherapy by inhibiting cell proliferation (45). Since the structure of BPTES has no similarity with glutamine, the off-target effect caused by the interaction with glutamine-related enzymes is reduced, and the toxic and side effects are small (56).

Compound 968 inhibits both GLS1 and GLS2. The mechanism of allosteric inhibition of GLS1 by compound 968 differs from that of BPTES, which is manifested by various binding sites and the most inhibition of GLS1 in the inactive state. Compound 968 inhibits the aberrant Rho-dependent signaling of GLS1 in tumor cells while having little effect on normal cell proliferation (58). Studies have shown that compound 968 inhibits the growth of ovarian cancer cells by inducing cell cycle arrest of G1-phase, cell apoptosis, and cellular stress, suggesting that targeting GLS1 is a therapeutic strategy for ovarian cancer (46). Wang et al. demonstrated that combination therapy with the GLS inhibitor 968 and PD-L1 blockade enhanced the immune response to ovarian cancer. *In vivo* experiments showed that compound 968 increased the infiltration of CD3+ T cells into ovarian cancer and enhanced the secretion of CXCL10 and CXCL11 by ovarian cancer cells (59). This research indicated the clinical significance of compound 968 combining immune checkpoint inhibitors in treating ovarian cancer.

CB-839 is an oral small-molecule GLS inhibitor developed based on BPTES (56). In a phase I clinical trial, CB-839 alone has a small inhibitory effect on most tumors. However, it is significantly inhibited when combined with other antitumor drugs. Multiple Phase I/II clinical trials have shown that CB-839 has well patient tolerance. It can effectively improve the drug resistance of paclitaxel, carboplatin, and everolimus, and enhance the effect of capecitabine, cabozantinib, etc., in inhibiting glutamine-dependent tumors. Treatment of ovarian cancer mice using CB-839 prolongs the survival of the mice (47). Studies have shown synergistic effects of CB-839 in combination with immune checkpoint inhibitor anti-PDL1 in an Arid1a-inactivated mouse ovarian cancer xenograft model (60). On ClinicalTrials.gov, we retrieved a CB-839 clinical trial combined with niraparib for patients having platinum-resistant ovarian cancer (NCT03944902). However, this study has been terminated and will not be resumed due to the company’s decision to stop using this drug. One participant has withdrawn from the study (See **Table 2**).

**Table 2:** Clinical trials of glutamine metabolism-related drugs and ovarian cancer.

NCT identifier	Study phase	Condition or disease	Treatments	Primary end point	Status
NCT05039801	I	Various solid tumors including ovarian cancer	Drug: Glutaminase Inhibitor IPN60090 Biological: Pembrolizumab	Incidence of AEs	Recruiting
NCT03944902	I	Ovarian Cancer Resistant BRCA Wild-Type Ovarian Cancer	Drug: CB-839 Dose Escalation	Access toxicity as evidenced by the number and percent of treatment AEs.	Terminated

AEs, adverse events.

GLS-specific inhibitors alone have no apparent inhibitory effect on tumors, and the search for drugs that broadly inhibit glutamine metabolism has attracted more attention. DON (6-diazo-5-oxo-norleucine) competitively binds to the active site of glutamine, forming a covalent compound that irreversibly inhibits various metabolic enzymes that utilize glutamine, such as GLS, GS, and transaminase. It has analgesic, antibacterial, antiviral, and tumor-inhibiting effects. However, its wide range of effects has strong toxic and side effects (61). Studies have shown that DON reduces the peritoneal adhesion of ovarian cancer in mice by inhibiting hyaluronan synthase activity (62). Recently, there have been many breakthroughs in the design of DON drugs. Prodrugs not metabolized in plasma but decomposed in target cells are obtained by modifying the carboxyl and amino groups of DON, including DON substituted with acetyl-lysine and JHU-083. *In vitro* and *in vivo* experiments showed that DON substituted with acetyl-lysine selectively metabolized in P493B lymphocytes and inhibited the proliferation of tumor cells in a dose-dependent manner (63). However, there are no relevant reports on ovarian cancer, and we look forward to furthering research.

##### 3.4.1.3 Cystine/glutamate transporter cystine-glutamate exchange (xCT) inhibitors

xCT promotes the synthesis of GSH and plays an important role in maintaining intracellular redox balance. Its inhibitors include sulfasalazine, erastine (Ras selective lethal small molecule 3), and sorafenib (64). The xCT-GSH/glutathione peroxidase 4 (glutathione peroxidase 4, GPX4) axis is the main signaling pathway of ferroptosis (65). These inhibitors induce ferroptosis, which may be an innovative approach to tumor therapy. The effect of sulfasalazine on glutathione consumption was examined in a preclinical study using an ovarian cancer model (52). Erastin is a novel specific small molecule that induces ferroptosis in ovarian cancer cells. Erastin and docetaxel act synergistically in ovarian cancer. The co-delivery of erastin with docetaxel can lead to significant cell viability reduction apoptosis, as well as cell cycle arrest in G2/M in ovarian cancer cells. This suggests that the combination of erastin and docetaxel may provide an effective drug delivery method for chemotherapy-resistant patients with ovarian cancer (48). Erastin also acts synergistically with cisplatin to inhibit ovarian cancer cell growth. This is completed possibly through a ROS-mediated mechanism to enhance the cisplatin effect (49). Sorafenib is a nonselective multikinase inhibitor with proven antiproliferative effects in thyroid, renal and hepatocellular carcinoma. There has been multiple phases I, II, and III clinical trials in ovarian cancer related to sorafenib. In several trials of sorafenib, the effects of sorafenib as a single agent or in combination with other chemotherapeutic agents have generally been modest, with complete responses rarely observed (50). In a subsequent phase II clinical trial combining sorafenib with bevacizumab in patients with recurrent ovarian cancer, although some clinical responses were observed, the prespecified primary endpoint was not met (51).

#### 3.4.2 Chemotherapy resistance of Gln metabolism in ovarian cancer treatment

Chemotherapy resistance is an important issue in the current drug treatment of ovarian cancer. A major pathogenic factor that results in cancer resistance to standard therapies is oncogenic heterogeneity. Various metabolic phenotypes may arise due to gene expression among different types of cancer, different patients with the same tumor type, and even within a single tumor. This makes a single-treatment approach insufficient (66). Platinum and paclitaxel are first-line chemotherapy drugs for ovarian cancer. Although 80% of ovarian cancer patients are sensitive to platinum-based first-line chemotherapy, the recurrence rate is as high as 60%. Most recurrent ovarian cancer patients would have platinum resistance and an extremely poor prognosis (67, 68). Studies have confirmed that c-myc regulates the dependence of Gln metabolism in platinum-resistant ovarian cancer cell lines through the tricarboxylic acid cycle and oxidative phosphorylation. At present, taking metabolic pathways as research targets to enhance the therapeutic efficacy of ovarian cancer and overcome drug resistance is a more attractive research direction. In the platinum-sensitive ovarian cancer cell line A2780, the metabolism of glucose and Gln is up-regulated in platinum drug treatment initially. In contrast, the platinum-resistant cell line CP70 is significantly dependent on Gln, accompanied by up-regulation of Gln transporters ASCT2 and GLS. The important role of Gln metabolism in causing platinum resistance was confirmed by stable overexpression of GLS. Down-regulation of GLS expression using small hairpin RNA (shRNA) in platinum-resistant ovarian cancer cells can resensitize the cells to platinum therapy. Significantly, the combination of GLS inhibitor (BPTES) and platinum can synergistically inhibit platinum-sensitive and resistant ovarian cancer *in vitro*. The combination of platinum and BPTES significantly increased the induction of tumor cell apoptosis compared with platinum alone. Therefore, combining the Gln metabolic pathway and platinum-based chemotherapy is a potential therapeutic strategy for ovarian cancer, especially for drug-resistant ovarian cancer (69).

Studies have shown that periodic fasting can improve the chemosensitivity of cancer cells (70). Florine et al. demonstrated for the first time the specific killing effect of periodic fasting on platinum-resistant tumor cells (71). Gln could inhibit the death of starved ovarian cancer cells, and depletion of Gln could resensitize platinum-resistant ovarian cancer cells. Various platinum-resistant cells can promote oxidative phosphorylation and upregulate the activities of Gln transporters ASCT2 and GLS by increasing glycolysis to boost Gln metabolism (69). Most studies have shown that the critical target of platinum resistance related to glutamine metabolism is GLS; knock out of GLS can resensitize platinum-resistant ovarian cancer cells to cisplatin, and overexpression of GLS can lead to platinum resistance (69). However, Florine et al. found that the inhibition of GLS by genes or drugs cannot induce the death of platinum-resistant cells (71). On the contrary, inhibition of GLS can induce the proliferation of platinum-resistant cells, which may be caused by reducing the conversion of intracellular Gln to glutamate to make Gln maintain at a level compatible with promoting nucleotide biosynthesis. Mass spectrometry metabolomics and specific interventions on glutamine metabolism showed that in cisplatin-resistant cells, Gln was mainly used for nucleotide biosynthesis rather than tricarboxylic acid replenishment, bioenergy, and redox reactions. Tardito et al. showed that the goal of inhibiting the death of Gln-deficient platinum-resistant glioblastoma cells could not be achieved by promoting the tricarboxylic acid cycle (72). According to Florine et al., ribonucleotide supplementation can effectively inhibit starvation-induced death of platinum-resistant lung cancer cells (71).

In conclusion, the above studies suggest that Gln promotes platinum resistance by increasing the concentration of intracellular nucleotides, rather than by promoting the tricarboxylic acid cycle. Therefore, cisplatin-resistant cancers become hypersensitive to periodic fasting and antimetabolite therapy targeting nucleotide metabolisms (73), such as the thymidine synthase inhibitor 5-fluorouracil (5-FU) and ribonucleic acid reductase inhibitor clofacitabine. It has been reported that compared with cisplatin alone in the treatment of malignant pleural mesothelioma, the combination of cisplatin and rititrexed can improve the overall survival rate (74), indicating that platinum chemotherapy drugs combined with anti-nucleotide metabolizers can increase the platinum sensitivity of ovarian cancer cell population.

## 4 Future directions

Although significant progress has been achieved in diagnosing and treating ovarian cancer in recent years, the development of prognosis is still poor. Gln catabolism contributes to the growth and invasion of ovarian cancer. Glutamate metabolism plays a crucial role in the diagnosis, development, and treatment of ovarian cancer. Theoretically intervening with one or more enzymes or genes of Gln metabolism can block the metabolism in ovarian cancer cells and achieve better therapeutic effects. For better treatment, in-depth experimental studies on Gln metabolism are required to determine whether Gln metabolism-blocking targeted medications affect normal cell metabolism and which drugs can prevent chemical drug resistance. Current studies have focused on overcoming the tumor’s adaptive changes after inhibiting glutamine metabolism, such as inhibiting multiple metabolic pathways at the same time (75). GLS inhibitor compounds 968, CB-839, SLC1A5 inhibitor V-9302, and monoclonal antibodies have significant antitumor activity in preclinical studies, and more clinical trials are urgently needed. DON and its derivatives inhibit the glutamine metabolism of tumor cells and increase the immune function of CD8^+^ T cells simultaneously. They are expected to become complementary therapy to immunotherapy. Since the role of Gln in tumor therapy is affected by various factors such as tissue type, genetics, tumor microenvironment, diet, and physiology, the individualized drug delivery for patients with glutamine-dependent tumors also needs to be further studied (9). There are few clinical trials of Gln metabolism inhibitors in ovarian cancer. One of the studies was terminated (**Table 2**). We look forward to more clinical studies related to Gln metabolism.

## 5 Conclusion

Ovarian cancer often occurs in perimenopausal women. Due to the lack of apparent symptoms and effective diagnosis in the early stage, its mortality rate always ranks first in gynecological tumors. In the past decade, tumor metabolomics research has reattracted researchers’ attention. It has been nearly a century since researchers discover the characteristics of tumor metabolism changes. Using new biochemical and molecular biological methods to study tumor metabolism will help increase the understanding of the mechanism and function of tumor-related metabolic changes in different periods. Gln, the second largest source of tumor nutrients, has become a new direction of ovarian cancer metabolism research. The above studies show that Gln catabolism contributes to the growth and invasion of ovarian cancer cells. Inhibiting Gln catabolism enzymes such as GLS can effectively block cancer invasion and make platinum-resistant ovarian cancer cells re-sensitize to platinum-based chemotherapy drugs. Therefore, targeting the Gln catabolic pathway is of great significance in searching for diagnostic and prognostic markers and new therapeutic targets for ovarian cancer.

## Author contributions

XY and JF take responsibility for the integrity of the data and the accuracy of the data analysis. XY and ZL contributed equally to this study. Concept and design: XY. Drafting of the manuscript: XY and ZL. Critical revision of the manuscript for important intellectual content: JF. Obtained funding: XY and JF. Technical support: XP. All authors contributed to the article and approved the submitted version.

## Funding

This study was funded by the Shanghai Jiao Tong University Affiliated Sixth People’s Hospital (contract grant number: ynqn202118) and the Science and Technology Project of Shanghai Municipal Science and Technology Commission (No. 22Y31900500). The funding organizations had no role in the design and conduct of the study; collection, management, analysis, and interpretation of the data; preparation, review, or approval of the manuscript; or decision to submit the manuscript for publication.

## Conflict of interest

The authors declare that the research was conducted in the absence of any commercial or financial relationships that could be construed as a potential conflict of interest.

## Publisher’s note

All claims expressed in this article are solely those of the authors and do not necessarily represent those of their affiliated organizations, or those of the publisher, the editors and the reviewers. Any product that may be evaluated in this article, or claim that may be made by its manufacturer, is not guaranteed or endorsed by the publisher.
